# Joubert syndrome gene *fam149b1* homolog, *xbx-4* , is required for multiple sensory behaviors.

**DOI:** 10.17912/micropub.biology.002141

**Published:** 2026-04-15

**Authors:** Kaitlin Ching, Paul W. Sternberg, Maureen M. Barr

**Affiliations:** 1 Genetics Department, Rutgers, New Brunswick, NJ, US; 2 Division of Biology and Biological Engineering, California Institute of Technology, Pasadena, CA, US

## Abstract

Many cells require cilia to receive environmental signals. Mutations in the ciliary gene *fam149b1* result in the ciliopathy Joubert syndrome. The *C. elegans* homolog of *fam149b1* , *xbx-4* , is required for normal cilium structure. We found that loss of XBX-4 hinders multiple cilium-mediated behaviors. *xbx-4* mutants display mild defects in male mating and nose touch behaviors and dramatic changes in social feeding. Unexpectedly, *xbx-4* mutants increased reversal behavior in response to ethanol, a control stimulus used in olfactory assays. Variation in the magnitude of phenotypes for different neurons is consistent with known cell-specific impacts of XBX-4 loss on cilium structure.

**Figure 1. Behavioral assays with worms lacking XBX-4:** (A) Schematic and plot of mating response efficiency for wild-type (WT) and *xbx-4* mutant males. Each dot represents one worm. n = 9 worms per genotype across N = 3 experiments. p = 0.0063 by Mann-Whitney U test. (B) Schematic and plot of nose touch responses for WT and *xbx-4* mutant hermaphrodites. Each dot represents one experiment. n = 40 worms per genotype across N = 5 experiments. Z = 3.9 by two-portion Z test. (C) Schematic and plot of olfactory avoidance in response to ethanol (EtOH control) or octanol diluted in ethanol (oct in EtOH, aversive stimulus) for WT and *xbx-4* mutant hermaphrodites. Each dot represents one worm. n = 16 worms per genotype across N = 3 experiments. p = 0.00090 (WT EtOH vs. WT oct) and 0.0049 (WT EtOH vs. *xbx-4* EtOH) by Mann-Whitney U test. (D) Schematics for two social feeding behaviors in WT (negative control), *xbx-4* mutant, and *osm-5* mutant (positive control) hermaphrodites and table for portion of each population displaying that behavior. n = number of worms for given genotype across N = 2 plates. Border dwelling: Z = -11.6 (WT vs. *xbx-4* ), -5.7 (WT vs. *osm-5* ), -6.8 ( *osm-5* vs. *xbx-4* ). Contact: Z = -8.7 (WT vs. *xbx-4* ), -3.8 (WT vs. *osm-5* ), -4.2 ( *osm-5* vs. *xbx-4* ) by two-portion Z test. For all plots, lines show mean values, and error bars show 95% confidence intervals. Asterisks indicate statistical significance (p < 0.05 or Z < -1.96 or > 1.96), including multiple hypothesis correction for D (Z < -2.4).

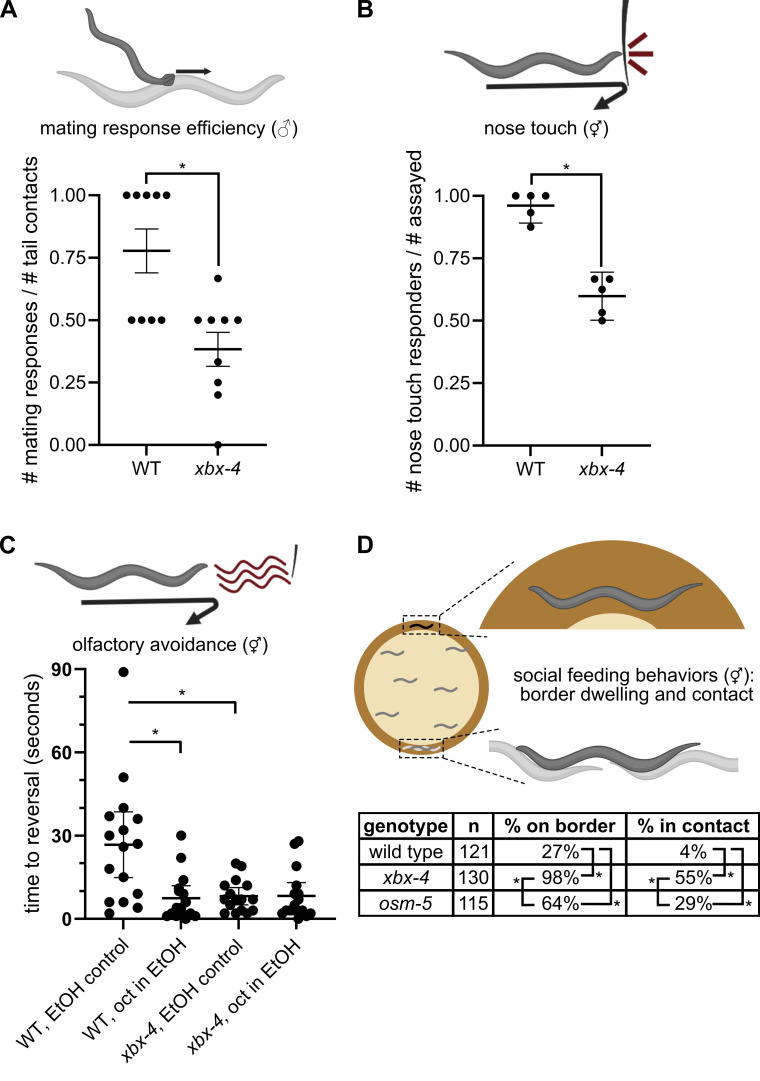



## Description

Cilia are hair-like organelles with a microtubule core that can serve as cellular antennae to receive environmental signals. Diseases resulting from ciliary dysfunction are known as ciliopathies. Mutations in ciliary protein FAM149B1 correspond with elongated cilia and disrupted Sonic Hedgehog (SHH) signaling, resulting in the ciliopathy Joubert syndrome (Shaheen et al., 2019). Whether changes to ciliary signaling are due to specific defects in SHH signaling or broader defects in ciliary function remains unclear. Nematodes lack a canonical Hedgehog-Patched-Smoothened signaling pathway (Bürglin and Kuwabara, 2005). Therefore, we sought to determine if the *Caenorhabditis elegans ( C. elegans ) fam149b1* homolog is involved in ciliary signaling.

The *C. elegans* *fam149b1* homolog *xbx-4* regulates cilium shape and length in AWA amphid wing sensory neurons (Maurya and Sengupta, 2021). *xbx-4* is expressed in dye filling ciliated amphid and phasmid sensory neurons in the head and tail (Maurya and Sengupta, 2021). The only ciliated cells in the *C. elegans* body are sensory neurons, and many of these neurons mediate well-characterized behaviors. To test the role of FAM149B1/ XBX-4 , we used the null allele *xbx-4 ( ok635 )* (Maurya and Sengupta, 2021). In this study, we assessed ciliary functions in *xbx-4* mutant animals using a variety of behavioral assays. We found that cilium function was, indeed, defective, though the severity of the sensory defect varied between the neuron types tested.

The first cilium-mediated behavior we assayed was mating. *C. elegans* populations include two sexes: self-fertile hermaphrodites and males, which can transfer sperm to hermaphrodites for reproduction. Male mating consists of a series of specific behaviors, which are mediated by specific sets of neurons (Liu and Sternberg, 1995). The first step, the mating response, occurs when the male tail contacts the hermaphrodite body. The tail flattens, and the male swims in reverse to scan the hermaphrodite body. Response behavior requires nine pairs of ray sensory neurons in the tail and requires functional cilia on these ray neurons (Barr and Sternberg, 1999; Barr et al., 2018). CeNGEN single-cell RNAseq data (Hammarlund et al., 2018) show that ray neurons contain high levels of *xbx-4* RNA.

To determine if XBX-4 is required for male mating behavior and ray cilium function, we placed males with *unc-31 ( e169 )* uncoordinated hermaphrodites, which move very little. We quantified male mating responses and normalized the number of responses to the number of times the male tail contacted a hermaphrodite. We found that *xbx-4* mutant males were significantly less efficient in their mating response compared to wild-type males (mean efficiency = 0.78 for wild type vs. 0.38 for *xbx-4* mutants, n = 9 worms per genotype across N = 3 experiments, p = 0.0063 by Mann-Whitney U test) ( **figure panel A** ). Most *xbx-4* mutant males exhibited a mating response and located the hermaphrodite vulva within 5 minutes (n = 8 out of 9), in contrast to severe cilium structure mutants (Barr and Sternberg, 1999). This reduced response efficiency suggests that the ciliated ray neurons are partially defective, but signaling is not completely abolished in the absence of XBX-4 .

To determine if cilium defects extend beyond ray neurons, we assayed the nose touch response because 79% of the behavior relies on the ciliated neurons ASH, FLP, and OLQ (Kaplan and Horvitz, 1993). We found that *xbx-4* mutant hermaphrodites had a reduced nose touch response (95% of wild type respond vs. 58% *xbx-4* mutants respond, n = 40 worms per genotype across N = 5 experiments, Z = 3.9 by two-portion Z test) ( **figure panel B** ). A subset of worms was retested three times in succession, and all worms were able to reverse at least once (n = 6). The contribution of non-ciliated neurons (ALM, AVM) accounts for only a small portion of the nose touch response (Kaplan and Horvitz, 1993), suggesting that the function of ciliated neurons ASH, FLP, and/or OLQ is defective in worms that lack XBX-4 .

To test a cilium-mediated behavior that is not mechanosensory, we assayed octanol avoidance. Octanol is an aversive olfactory stimulus that relies primarily on ciliated ASH neurons for short-range chemosensation (Troemel et al., 1995). Wild-type hermaphrodites reversed more quickly when presented with 10% octanol than with the ethanol control (27 seconds for ethanol vs. 7.4 second for octanol, n = 16 worms per genotype across N = 3 experiments, p = 0.00090 by Mann-Whitney U test). Surprisingly, *xbx-4* mutants reversed significantly faster than wild-type animals when presented with the ethanol control stimulus (27 seconds for wild type vs. 8.2 seconds for *xbx-4* mutants, p = 0.0049 by Mann-Whitney U test) ( **figure panel C** ). Whether the decreased response time is due to increased ethanol sensation, which may be mediated by IL2 ciliated neurons (Johnson et al., 2017), or simply a higher baseline rate of reversal requires further investigation. The high reversal rate of *xbx-4* mutants in the ethanol control condition made it difficult to determine if *xbx-4* mutants had a defect in olfactory avoidance. However, the high rate of reversal suggests that the defective mating and nose touch responses, which both require worms to swim backward, were not due to a decreased ability to reverse. Instead, the high reversal rate confirms that decreased mating and nose touch responses were specific to the function of the ciliated neurons.

While conducting other behavioral assays, we noted that *xbx-4* mutant worms had an increased tendency to reside in groups at the edge of the bacterial lawn, so we assayed social feeding behaviors. Social feeding is observed in some isolates of *C. elegans* ; however, the lab strain Bristol N2 isolate typically does not exhibit this behavior due to the inhibitory function of ciliated AQR and PQR neurons, as well as the non-ciliated URX neuron (de Bono and Bargmann, 1998; Coates and de Bono, 2002). *xbx-4* mutant hermaphrodites were significantly more likely to dwell on the border of the bacterial lawn on which they feed (27% of 121 wild-type animals, 98% of 130 *xbx-4* mutants across N = 2 plates each, Z = -11.6 by two-portion Z test). *xbx-4* mutants were also more frequently in contact with at least one other worm along the majority of the body (4% of wild type vs. 55% of *xbx-4* mutants, Z = -8.7 by two-portion Z test) ( **figure panel D** ). Because the phenotype was so dramatic, we also included worms lacking the well-characterized cilium structure gene *osm-5* /IFT88. We found that *xbx-4* mutant worms had stronger social feeding behaviors than *osm-5* mutant worms, which have defective cilium structure and function (64% on border and 29% in contact of 115 *osm-5* mutant animals, Z = -4.2 by two-portion Z test) ( **figure panel D** ). These strong social feeding behaviors are consistent with a severe defect in AQR and PQR cilia in worms that lack XBX-4 , with the caveat that function of the non-ciliated URX neuron in *xbx-4* mutants is not known.

Taken together, results from our behavioral assays demonstrate that worms lacking XBX-4 are defective in multiple cilium-mediated sensory behaviors to varying degrees. *xbx-4* mutants exhibited mild defects in male mating and nose touch responses, a striking increase in cilium-opposing social feeding behaviors, and an unexpected increase in reversal rate in our control olfactory condition (ethanol). These results suggest that ciliary signaling defects observed in cells from Joubert syndrome patients with mutated *fam149b1* may not be specific to the SHH pathway and may extend to ciliary functions more broadly. One model consistent with our findings is that sensory neurons rely on XBX-4 in a cell-type specific manner, depending on the structure, environment, or composition of the cilium. This model is supported by the finding that the wing-shaped cilia of AWA neurons is dramatically changed in *xbx-4* mutants, whereas the rod-shaped cilia of ASH amphid channel neurons exhibit a subtle but significant change in length (Maurya and Sengupta, 2021). It is not known if ciliary signaling changes in *xbx-4* mutants are due to ciliary ultrastructural defects, specific cellular environments, and/or specific composition of different cilia. Although our findings are consistent with the predicted role of XBX-4 , a caveat is that we only examined one *xbx-4* mutant allele. Phenotypes observed could be due to an unrelated mutation, and examination of additional mutant lines or rescue of the *xbx-4 ( ok635 )* mutant phenotypes with wild-type *xbx-4 (+)* would be needed to rule out this possibility. Further resolving the function of *xbx-4* may shed light on the connection between cilium structure and signaling and the underlying disease mechanism for Joubert patients with disease-causing variants of *fam149b1* .

## Methods

*Strains and conditions*

Animals were cultured at 20°C on standard nematode growth medium (NGM) with the OP50 strain of *E. coli* bacteria as a food source (Brenner, 1974). For strains lacking a *him-5* mutation, males were initially obtained by heat shocking L4 hermaphrodites (30°C for 5.25-7.5 hours), picking male progeny, and maintaining plates by crossing males and hermaphrodites. Note that, due to reduced mating efficiency, *xbx-4* mutants generally required more males, relative to N2 , to maintain enough male progeny for experiments. For each experimental replicate, an equal number of wild type or mutant animals were assayed on plates from the same batch of NGM and OP50 . This study used the strains listed in Table 1.

Table 1: strains used in this study

**Table:** 

**genotype**	**source, strain code, and reference**
wild type	Caenorhabditis Genetics Center (CGC) ( N2 )
*xbx-4 ( ok635 ) *	CGC ( OE3003 )
*osm-5 ( sa126 ); him-5 ( e1490 ) *	Barr lab (PT40 (Qin et al., 2001))
*unc-31 ( e169 ) *	Sternberg lab ( CB169 (Avery et al., 1993))

&nbsp;

*Male mating*

L4 *unc-31* mutant hermaphrodites were selected and grown overnight. Wild-type and mutant L4 males were selected and assayed the next day (24-27 hours later) with the observer blinded to the genotype. Assay plates were standard NGM plates seeded with 10 µL of fresh OP50 grown for 2 hours at 37°C and then cooled to room temperature. *unc-31* mutant hermaphrodites were allowed to settle on the plate for at least ten minutes prior to adding males. A new plate of hermaphrodites was used after assaying approximately four males. A mating response was scored if the tail of the male flattened against the hermaphrodite and the male swam in reverse, scanning the hermaphrodite (Liu and Sternberg, 1995). The number of mating responses was normalized to the number of times the male's tail contacted a hermaphrodite, resulting in a response efficiency score for each animal.

&nbsp;

*Nose touch*

Wild-type and mutant L4 hermaphrodites were selected and assayed the next day as adults with the observer blinded to the genotype (22-24 hours later) on standard NGM plates that had been seeded with 120 µL OP50 grown overnight. Each worm was assayed individually by placing a clean eyebrow pick in front of the forward-swimming worm and waiting for contact. A reversal response was scored if the animal immediately reversed upon contact, and a failure was scored if regions at or posterior to the pharyngeal bulb made contact before reversal, typically occurring if the hermaphrodite swam over, under, or along the side of the eyebrow.

&nbsp;

*Olfactory avoidance*

Wild-type and mutant L4 hermaphrodites were selected and assayed the next day (24 hours later) with the observer blinded to the genotype. Each animal was assayed on a standard NGM plate that had been seeded with 120 µL of OP50 grown overnight or briefly at 37°C, resulting in a thin lawn. Each animal was scored first with the ethanol (control) stimulus and then with 10% 1-octanol (Tokyo Chemical Industry Products #O003625ML) dissolved in ethanol. For each test, an eyebrow pick was dipped in the solution for approximately 5 seconds and then immediately held in front of the forward-swimming animal located in the center of the field of view. The stimulus was kept in front of the animal until it swam in reverse, defined as at least one body bend in the posterior direction. For animals that did not reverse, time to reversal was recorded as the time the animal swam out of the field of view. Robustness of the octanol response in wild-type worms varied greatly between replicates, possibly due to environmental conditions (e.g. temperature or air flow). Therefore, data from an experimental replicate were only included if a majority of wild-type worms responded to the octanol stimulus (determined after data were unblinded).

&nbsp;

*Social behaviors: border dwelling*

Wild-type and mutant hermaphrodite L4 animals were selected, genotypes blinded, and grown for two days. Assay plates were made by seeding standard NGM plates with 120 µL of fresh OP50 , incubating at 37°C for one hour, and then incubating at room temperature (20.5°C) overnight. Border thickness was visually determined to be similar for all plates used. For each genotype, a similar number of animals (50 for one replicate, 80 for the other) were picked onto each assay plate and incubated at 20°C for 2.5-3.5 hours. Number of animals in contact with the border, defined as the dark edge of the OP50 lawn, and the total number of live animals remaining on the NGM (i.e. not dead on the plastic surfaces, as is common for *osm-5* mutants, which leave the NGM and desiccate on the walls and lids of the Petri dishes) were scored for each plate.

&nbsp;

*Social behaviors: contact*

The same plates used for the border dwelling assay were used to score body contact. At the end of incubation, the amount of the hermaphrodite's body in contact with another hermaphrodite was estimated visually. The number of animals with at least 50% of the body in contact with another animal was scored and normalized to the total number of animals remaining on the NGM.

&nbsp;

*Statistical methods*

For the male mating, nose touch, and olfactory avoidance assays, a small pilot data set was collected and used for a power analysis (power = 0.8) to determine the final size of the data set. For social behaviors, both replicates were conducted on the same day, and no additional data were collected. Plots were generated and Mann-Whitney U tests were conducted in GraphPad Prism (version 10.4.2). Two-portion Z tests were computed in Microsoft Excel.

&nbsp;

*Knowledge Bases*

WormBase was used throughout experimental planning and interpretation of results (Sternberg et al., 2024).
